# Use of Intraoperative Doppler Ultrasonography in Predicting Life-Threatening Vascular Complications After Adult Deceased Donor Liver Transplantation

**DOI:** 10.7759/cureus.73588

**Published:** 2024-11-13

**Authors:** Catherine O'Leary, Samantha Spence, Reeder M Wells, Daniel Sculley, Jefferey Bettag, Raymond I Okeke, Ramy Shoela, Mustafa Nazzal

**Affiliations:** 1 Department of Surgery, Saint Louis University School of Medicine, St. Louis, USA; 2 Department of Surgery, Sisters of St. Mary (SSM) Health Saint Louis University Hospital, St. Louis, USA; 3 Department of Radiology, Saint Louis University Hospital, St. Louis, USA; 4 Department of Surgery, Saint Louis University Hospital, St. Louis, USA

**Keywords:** flow abnormalities, intraoperative doppler ultrasound, liver transplantation, prevention, vascular complication

## Abstract

Aim

This study aims to determine if routine use of intraoperative Doppler ultrasonography is preventative of life-threatening vascular complications (VCs) after orthotopic liver transplantation.

Methods

This single-center, retrospective study reviewed all adult orthotopic liver transplants at Saint Louis University Hospital from 2015 to 2020 (N = 188). The sample population consists of men and women in the age range of 18 to 75. Operative reports were reviewed for the use of intraoperative ultrasound (IOUS) and the associated resistive indices, peak systolic velocities, and qualitative assessments of flow. Postoperative VCs were identified as complications requiring intervention between the time of transplant and December 31, 2020. Life-threatening VCs were defined by the presence of vascular thrombosis. The primary outcome was the incidence of postoperative life-threatening VCs between those in which intraoperative DUS was performed and those in which it was not.

Results

IOUS was documented in 35 (18.6%) cases. All cases using IOUS demonstrated good flow and no abnormalities, as reported by the operating surgeon. There was no difference in patient population between those who received IOUS and those who did not. Postoperative life-threatening VCs were identified in five cases. Of the cases in which no IOUS was performed, five (3.3%) had life-threatening VCs. Of the patients with documented IOUS, 0 (0%) had life-threatening VCs.

Conclusions

The IOUS group showed a lower incidence of life-threatening VCs (0%) compared to the no IOUS group, which had a 3.3% life-threatening complication rate. However, this was not statistically significant due to the small number of VCs, as VCs following liver transplants are inherently rare. With these results in combination with current literature, there is support for the use of IOUS in preventing and predicting VCs.

## Introduction

Postoperative vascular complications (VCs) in orthotopic liver transplantation (OLT) can have dire consequences, including graft failure and patient death, if not detected and treated early [1]. Though rare, VCs represent the most common cause of morbidity and mortality following liver transplant [2]. These adverse outcomes can arise due to issues with anastomotic technique, infection, or graft rejection [3] and can present days to years after transplant. VCs can be arterial or venous in origin, inflow or outflow related, and include stenosis, thrombosis, pseudoaneurysm, and rupture [4,5]. Arterial complications are the most common VCs, associated with increased morbidity, decreased patient survival, and subsequent complications such as biliary leak or stricture [6-8]. Hepatic artery thrombosis (HAT) represents over 50% of all arterial VCs [3]. HAT occurs in approximately 4-12% of liver transplantation cases [3] and has a mortality rate of over 40% [9]. HAT is typically an early complication and is the leading cause of a primary nonfunctional liver graft [10]. Post-transplant hepatic artery stenosis (HAS) has an incidence of approximately 2-13% [3]. Venous complications are less frequent than arterial complications but are nonetheless life-threatening. These include thrombosis and stenosis of the portal vein, hepatic veins, or inferior vena cava (IVC), each with a reported incidence of approximately 1-3% [11,12].

Doppler ultrasonography (DUS), along with angiographic modalities such as computed tomography angiography (CTA) and magnetic resonance angiography (MRA), can non-invasively assess vascular patency. DUS performed intraoperatively may allow surgeons to prevent VCs and/or identify patients at risk for developing VCs. This could decrease incidence rates and time to treatment and improve patient outcomes [13]. In recent years, literature has supported the use of intraoperative DUS for real-time evaluation of the anastomoses to identify and address VCs during the procedure [13-15] and predict their likelihood of occurring postoperatively [16,17]. The primary aim of this study is to determine if the use of intraoperative ultrasound (IOUS) decreases the incidence of life-threatening VCs postoperatively among those who received IOUS compared to those who did not.

## Materials and methods

All adult (≥18 years of age) liver transplants performed at Saint Louis University Hospital (St. Louis, Missouri, USA)between January 1, 2015 and December 31, 2020 were retrospectively reviewed. The sample included combined liver and kidney transplants as well as re-transplants. The Saint Louis University institutional review board approved the study (IRB #32285) and waived patient consent requirement due to the retrospective, de-identified nature of the study. Data collection took place between April 13, 2022, and March 15, 2023. Data analysis was conducted from March 20, 2023, to April 24, 2023. Operative reports were reviewed for the use of IOUS and the associated resistive indices, peak systolic velocities, and qualitative assessments of flow. 

Recipient demographics

Demographic and medical history data for each patient were recorded. Recipient demographic information collected included the sex, ethnicity, and race of each patient as well as their body mass index (BMI), age, and model for end-stage liver disease (MELD) score at the time of transplant. The patients were then further characterized by primary end-stage liver disease diagnosis, including alcohol (EtOH), hepatitis C virus (HCV), nonalcoholic steatohepatitis (NASH), primary sclerosing cholangitis (PSC), or primary liver malignancy. Lastly, it was noted whether this was a primary transplant or re-transplant and if the donated organ was hepatitis C positive.

Donor characteristics

The donor type was reviewed for donation after brain death (DBD) or donation after cardiac death (DCD), and all split liver grafts were noted.

Operative technique for arterial anastomosis and implantation of liver

Variables for analysis regarding operative techniques included the type of arterial reconstruction for inflow and the type of hepatic vein reconstruction. This included the use of either the recipient's hepatic artery or a jump graft from the aorta. For hepatic vein reconstruction, recipients underwent either a piggyback technique or bicaval conventional. 

Use of IOUS in liver transplant 

The primary aim of this study was to determine the effect of IOUS use on the incidence of VCs postoperatively. Operative reports were reviewed for any documented use of IOUS and associated sonographic findings. All available IOUS reports were reviewed, and any intraoperative flow abnormalities were documented. A normal IOUS was defined as a hepatic artery resistive index (RI) of 0.55-0.8 and acceleration time of <80 ms, triphasic waveforms visualized in the hepatic veins, and portal vein hepatopetal flow with a velocity of >10 cm/s. Any deviation from normal flow values was considered an abnormal IOUS. All flow abnormalities were documented as well as any post-evaluation interventions performed during the case.

Postoperative VCs

Postoperative VCs were determined by identified complications requiring intervention or re-transplant at any point between the time of transplant and December 31, 2020. Each VC was further categorized as either life-threatening or non-life-threatening, as this largely dictates the urgency of diagnosis and treatment intervention. Life-threatening complications were defined by the presence of vascular thrombosis or those leading to patient death, while other non-life-threatening VCs, defined by the absence of thrombosis, such as HAS requiring intervention, were reviewed but were not included in data analysis. 

Statistical analysis

Statistical analysis was performed using jamovi (the jamovi project, Jonathon Love, Damian Dropmann, and Ravi Selker, Sydney, Australia), a free and open statistical software built on top of the R statistical language (R Foundation for Statistical Computing, Vienna, Austria). Categorical variables were analyzed using chi-squared tests. Fischer's exact test was used when cell counts were too low for the use of a chi-squared test. Continuous variables were analyzed using Student's t-tests. Mann-Whitney U tests were used when a variable was not normally distributed.

## Results

Of the 200 OLTs performed between 2015 and 2020, 188 had operative reports that were available for review and met the inclusion criteria. 

Demographics

This study population was predominantly male (132, 70.2%, N = 188) and White (173, 92%, N = 188), with a mean age of 57.5 years and a BMI of 28.2. Of the cases, 182 (96.8%, N = 188) were primary transplants, with only six (3.2%, N = 188) being re-transplants. Alcoholic cirrhosis (53, 28.2%, N = 188) and chronic HCV (49, 26.1%, N = 188) were the most common causes of end-stage liver disease, indicating transplant. The average MELD score was 24.3. In all cases, there were no significant differences in patient population between patients who experienced VCs versus those who did not and between patients who received IOUS and those who did not (Table 1). 

**Table 1:** Descriptive statistics The data for variables including vascular complications, sex, ethnicity, race, hepatitis C organ, re-transplant, type of arterial construction, type of liver implantation, and indication for transplant have been represented as N (%). The data for age is represented as median (interquartile range), minimum, and maximum. The data for BMI and model for end-stage liver disease (MELD) score are represented as mean ± SD. p- values < 0.05 were considered statistically significant; however, none of the variables met this significance threshold. *Chi-squared test; **Fisher's exact test; ***t-test; ****Mann-Whitney U test

Variable	Total (N = 188)	Use of intraoperative ultrasound		p-value	Statistical test value
		Yes (N = 35)	No (N = 153)		
Vascular complications				0.586**	-
Yes - life-threatening	5 (2.7)	0 (0.0)	5 (3.3)	-	-
No	183 (97.3)	35 (100)	148 (96.7)	-	-
Sex				0.86*	0.0304
Female	56 (29.8)	10 (28.6)	46 (30.1)	-	-
Male	132 (70.2)	25 (71.4)	107 (69.9)	-	-
Ethnicity				1**	-
Hispanic/Latino	1 (0.5)	0 (0.0)	1 (0.6)	-	-
Not Hispanic/Latino	187 (99.5)	35 (100)	152 (99.4)	-	-
Race				0.3**	-
White	173 (92)	34 (97.1)	139 (90.8)	-	-
African American	10 (5.3)	0 (0.0)	10 (6.5)	-	-
Asian	3 (1.6)	1 (2.9)	2 (1.3)	-	-
Unknown	2 (1.1)	0 (0.0)	2 (1.3)	-	-
Hepatitis C organ				0.75**	-
Yes	18 (9.6)	4 (11.4)	14 (9.2)	-	-
No	170 (90.4)	31 (88.6)	139 (90.8)	-	-
Re-transplant				1**	-
Yes	6 (3.2)	1 (2.9)	5 (3.3)	-	-
No	182 (96.8)	34 (97.1)	148 (96.7)	-	-
Type of arterial construction				1**	-
Aortic anastomosis	5 (2.7)	1 (2.9)	4 (2.6)	-	-
Hepatic artery anastomosis	183 (97.3)	34 (97.1)	149 (97.4)	-	-
Type of liver implantation				0.98*	0.000485
Bicaval	54 (28.7)	10 (28.6)	44 (28.8)	-	-
Piggyback	134 (71.2)	25 (71.4)	109 (71.2)	-	-
Indication for transplant				0.21*	7.22
Alcoholic cirrhosis	53 (28.2)	13 (37.1)	40 (26.1)	-	-
Hepatitis C cirrhosis	49 (26.1)	5 (14.3)	44 (28.8)	-	-
Metabolic-associated fatty liver disease	41 (21.8)	10 (28.6)	31 (20.2)	-	-
Other	28 (14.9)	4 (11.4)	24 (15.7)	-	-
Primary sclerosing cholangitis	10 (5.3)	3 (8.6)	7 (4.6)	-	-
Primary liver malignancy	7 (3.7)	0 (0.0)	7 (4.6)	-	-
Age				0.96****	2661
	59.5 (53.3, 64.3), 19.1, 73	59.2 (53.8, 64.5), 32.3, 73	59.6 (53.2, 64.2), 19.1, 72.1	-	-
BMI				0.86***	-0.177
	28.2 ± 4.9	28.3 ± 4.5	28.2 ± 5.1	-	-
MELD score				0.55***	-0.592
	24.2 ± 8.6	24.7 ± 4.3	24.1 ± 9.3	-	-

Donor characteristics

All the donor livers in this sample were from donors after brain death, while no livers were donated after cardiac death. Eighteen (9.6%, N = 188) of donated livers were positive for hepatitis C. One patient received a split liver graft (0.53%, N = 188) (Table 1). 

Operative technique for arterial anastomosis and implantation of liver 

An aortic anastomosis was used in five (2.7%, N = 188) cases. Hepatic artery anastomosis was used in 183 (97.3%, N = 188) cases. A bicaval implantation method was used in 54 (28.7%, N = 188) cases. The piggyback method was used in 134 (71.2%, N = 188) cases (Table 1). 

Use of IOUS in liver transplant 

IOUS was documented in 35 (18.6%, N = 188) cases (Table 1). The use of IOUS was solely due to surgeon preference. All IOUS reports demonstrated good flow with no abnormalities requiring further intervention, as reported by the operating surgeon. 

Postoperative life-threatening VCs 

Postoperative life-threatening VCs were identified in five (2.7%, N = 188) cases (Table 1 and Table 2). Of the five patients who developed life-threatening VCs, 60% were female, and 40% were male. All patients who experienced VCs were White. The mean age was 63.1. The average BMI and MELD scores of these patients were 27.3 and 20, respectively. No VCs occurred in hepatitis C organs or in cases of re-transplantation. Of the life-threatening VC patients (N = 5), three (60%) received transplantation due to HCV cirrhosis, one (20%) for metabolic-associated fatty liver disease (MAFLD), and one (20%) for alcoholic cirrhosis. No VCs occurred in patients receiving transplants for primary liver malignancy or PSC. Three VC patients had piggyback implantation, while two had bicaval. All patients with life-threatening VCs had hepatic artery anastomoses (Table 1). IOUS was not performed in the five patients who later developed life-threatening VCs (Table 1).

**Table 2:** Postoperative vascular complications of liver transplantation IR, interventional radiology; IVC: inferior vena cava; HAS: hepatic artery stenosis; LFTs: liver function tests

Vascular complications	Life-threatening or non-life-threatening	Presentation	Diagnosis	Treatment
IVC thrombosis	Life-threatening	Asymptomatic; incidentally found on imaging	CT of the abdomen and pelvis	Heparin drip and exploratory laparotomy with revision of the suprahepatic caval anastomosis and thrombectomy of the IVC with subsequent IVC filter placement.
Hepatic artery thrombosis	Life-threatening	Increasing confusion, hypotension, and elevated bilirubin	Ultrasound + magnetic resonance angiography (MRA)	Mechanical thrombectomy of the recipient common hepatic artery, chemical thrombolysis (TPA), and bypass graft with an aorto-hepatic artery conduit. Eventual re-transplantation due to recurrent thrombosis.
Hepatic vein thrombosis	Life-threatening	Elevated LFTs	Triple-phase CT	Catheter directed thrombolysis + suction thrombectomy. Final resolution with venoplasty-anastomosis ballooning.
HAS/IVC thrombosis	Life-threatening	Incidental finding on imaging	HAS: ultrasound and angiogram; IVC thrombosis: IVC venogram	HAS: unsuccessful stenting and balloon angioplasty; IVC thrombosis: balloon venoplasty.
Portal vein thrombosis	Life-threatening	Incidental finding on imaging	US, MRI, CT, and spleno-portal venogram	TPA infusion catheter + angioplasty with stent placement.
Hepatic artery stenosis	Non-life-threatening	Nausea, vomiting, and elevated LFTs	CT phase 3 + ultrasound	IR angioplasty and heparin drip for 24 hours with resulting patency of vasculature.

Of the VC patients, one presented on postoperative day 1 with an incidental finding of an IVC thrombus extending from the renal vein to the anastomosis on CT imaging. The patient was treated via exploratory laparotomy with open thrombectomy of the IVC and revision of the suprahepatic caval anastomosis (Table 2). The next patient with a life-threatening VC was determined to have HAT on postoperative day 19, confirmed by DUS and MRA. During exploratory laparotomy, the hepatic artery anastomosis was found to have absent flow, which was addressed with both mechanical thrombectomy and chemical thrombolysis with reanastomosis. Despite confirmation of adequate arterial flow and thrill, the patient suffered from recurrent HAT, leading to liver re-transplantation (Table 2). A third patient was found to have hepatic vein thrombosis (HVT) on day 42, with a thrombus of the right hepatic vein extending to the intrahepatic IVC visualized on triple-phase CT. The patient underwent catheter-directed thrombolysis and balloon venoplasty with full resolution of the obstruction (Table 2). The fourth VC patient presented at two separate times with HAS and IVC thrombosis at postoperative days 6 and 18, respectively. Ultrasound and angiography revealed HAS and thrombosis of the IVC at the anastomosis between the native and transplant IVC. Despite the improvement of stenosis with balloon venoplasty, the patient continued to experience complications throughout their postoperative course, eventually resulting in the withdrawal of life-sustaining care and patient death (Table 2). The last life-threatening VC patient presented with PVT was found incidentally during follow-up imaging on postoperative day 96. At that time, MRI, CT, and DUS were concerning for chronic PVT, which was later confirmed by spleno-portal venogram. Treatment with tissue plasminogen activator (tPA) and angioplasty of the portal vein resulted in the resolution of the obstruction (Table 2). 

Although we did not include this patient in our data analysis, we did note one patient with a non-life-threatening VC. They presented on postoperative day 42 with HAS, diagnosed with triple-phase CT revealing stenosis and reduced RIs on DUS. Notably, IOUS at the time of transplant showed no flow abnormalities. The patient underwent hepatic artery angioplasty with improved vessel patency (Table 2).

## Discussion

Due to the high risk of morbidity and mortality from VCs following OLT, this study aimed to determine the effect of IOUS on the incidence of postoperative life-threatening VCs. Secondarily, the study aimed to evaluate the utility of IOUS in predicting VCs or identifying patients at higher risk of VC. Only 35 (18.6%, N = 188) cases utilized IOUS. In cases where IOUS was used, no flow abnormalities were noted. Of these cases, no patients suffered from a life-threatening VC (0%, N = 35). Alternatively, five (3.3%, N = 153) of the cases where IOUS was not utilized had life-threatening VCs (p = 0.586) (Table 1). Therefore, the IOUS group showed a lower life-threatening VC rate compared to those without IOUS. While it is possible there was abnormal flow that went undetected in the VC cases without IOUS, we are unable to determine if flow abnormalities are predictive of VCs as no patient with IOUS had abnormal flow patterns or developed life-threatening VCs. Lastly, no patient factors (e.g., prior comorbidities, reason for transplant, age) were identified in this cohort as risk factors for VCs. 

Despite this study not reaching statistical significance to evaluate the effect of IOUS on postoperative VCs, many studies have supported the use of IOUS. Someda et al. studied the changes in graft hemodynamics induced by VCs in liver transplant patients. When looking at 46 pediatric partial liver transplantation recipients who underwent IOUS, they found 12 with decreased portal venous flow, allowing the surgeons to correct the corresponding vascular abnormality intra-operatively [18]. In five patients, IOUS detected abnormal hepatic arterial flow, allowing for intra-operative correction with subsequent improvement of flow. Lastly, four of five venous outflow obstruction patients showed abnormal waveforms and flow velocity, which were subsequently corrected [18]. Dodd et al. determined that there was a statistically significant difference in the resistive indices and systolic acceleration times measured using DUS between patients with stenosis or thrombosis versus those with normal arteries [19]. These studies demonstrate the ability of IOUS to identify vascular flow abnormalities, which can be corrected at the time of transplant, preventing more serious, potentially life-threatening VCs in the postoperative course. Cheng et al. further demonstrated the utility of IOUS in a case series of nine patients with identified VCs using IOUS. The subsequent interventions resulted in surgical correction at the time of transplant and 100% graft survival [14]. They found that IOUS has not been shown to significantly increase operative times, is more succinct compared to postoperative DUS, and allows the surgeon to detect flow and anatomic abnormalities while still in the operating room [14]. While not yet fully characterized, the low financial and time cost of IOUS is likely to make its routine use worthwhile.

Our study’s major limitations included both small sample sizes and lack of randomization. The minimal number of patients receiving IOUS during liver transplantation may relate to the lack of standardization of its use. 

Further prospective studies with large sample sizes in which patients undergoing liver transplantation are randomized into IOUS and non-IOUS groups are needed to fully understand the role of IOUS after OLT in preventing life-threatening VCs. A larger meta-analysis of the existing single-center studies may be required to attain a sample size that is powered to look at postoperative complications with an incidence below 10%. 

## Conclusions

Although this study did not reach statistical significance due to a small number of VCs, as VCs following liver transplants are inherently rare, our results show a significantly lower rate of life-threatening VCs when IOUS is performed. Of note, all patients who underwent IOUS had a normal ultrasound at the time of transplant and did not have post-operative life-threatening VCs. All patients with post-operative life-threatening VCs did not have IOUS. Therefore, it is possible that there was an abnormal flow that went undetected in the VC cases. Additionally, we did not note any patient factors, including age, sex, method of transplantation, or reason for transplantation, as statistically significant risk factors for post-operative VCs. Our study found IOUS to be a safe, efficient, cheap method of evaluating vascular flow intra-operatively. Despite the lack of statistical significance, due to the combination of our findings and support from current literature, we believe in the benefit of performing IOUS. Further prospective studies and/or meta-analyses are needed to better evaluate the role of IOUS after OLT in preventing life-threatening VCs.

## References

[1] Li Q, Wang Y, Ma T (2021). Preoperative platelet count predicts posttransplant portal vein complications in orthotopic liver transplantation: a propensity score analysis. BMC Gastroenterol.

[2] Duffy JP, Hong JC, Farmer DG, Ghobrial RM, Yersiz H, Hiatt JR, Busuttil RW (2009). Vascular complications of orthotopic liver transplantation: experience in more than 4,200 patients. J Am Coll Surg.

[3] Wozney P, Zajko AB, Bron KM, Point S, Starzl TE (1986). Vascular complications after liver transplantation: a 5-year experience. AJR Am J Roentgenol.

[4] Namgoong JM, Hwang S, Ahn CS (2021). Indications and outcomes of liver transplantation for post-Kasai biliary atresia in young adults. Korean J Transplant.

[5] Pinto LE, Coelho GR, Coutinho MM, Torres OJ, Leal PC, Vieira CB, Garcia JH (2021). Risk factors associated with hepatic artery thrombosis: analysis of 1050 liver transplants. Arq Bras Cir Dig.

[6] Zhao JZ, Qiao LL, Du ZQ (2021). T-tube vs no T-tube for biliary tract reconstruction in adult orthotopic liver transplantation: an updated systematic review and meta-analysis. World J Gastroenterol.

[7] Hann A, Seth R, Mergental H (2021). Biliary strictures are associated with both early and late hepatic artery stenosis. Transplant Direct.

[8] Cherchi V, Vetrugno L, Zanini V (2021). Association between indocyanine green clearance test and ischemic type biliary lesions within one year after orthotopic liver transplantation. Gastroenterol Hepatol.

[9] Wu L, Zhang J, Guo Z (2011). Hepatic artery thrombosis after orthotopic liver transplant: a review of the same institute 5 years later. Exp Clin Transplant.

[10] Chen W, Tu Q, Zheng H (2021). An orthotopic liver transplantation patient survived without hepatic artery flow due to thrombosis: a case report. Transplant Proc.

[11] Wang SL, Sze DY, Busque S, Razavi MK, Kee ST, Frisoli JK, Dake MD (2005). Treatment of hepatic venous outflow obstruction after piggyback liver transplantation. Radiology.

[12] Delgado-Moraleda JJ, Ballester-Vallés C, Marti-Bonmati L (2019). Role of imaging in the evaluation of vascular complications after liver transplantation. Insights Imaging.

[13] Cheng YF, Chen CL, Huang TL (2004). Risk factors for intraoperative portal vein thrombosis in pediatric living donor liver transplantation. Clin Transplant.

[14] Cheng YF, Huang TL, Chen CL (1998). Intraoperative Doppler ultrasound in liver transplantation. Clin Transplant.

[15] Felsted AE, Shi Y, Masand PM, Nuchtern JG, Goss JA, Vasudevan SA (2015). Intraoperative ultrasound for liver tumor resection in children. J Surg Res.

[16] Choi JY, Lee JY, Lee JM, Kim SH, Lee MW, Han JK, Choi BI (2007). Routine intraoperative Doppler sonography in the evaluation of complications after living-related donor liver transplantation. J Clin Ultrasound.

[17] Gu LH, Fang H, Li FH, Li P, Zhu CX, Zhu JJ, Zhang SJ (2012). Prediction of early hepatic artery thrombosis by intraoperative color Doppler ultrasound in pediatric segmental liver transplantation. Clin Transplant.

[18] Someda H, Moriyasu F, Fujimoto M (1995). Vascular complications in living related liver transplantation detected with intraoperative and postoperative doppler US. J Hepatol.

[19] Dodd GD 3rd, Memel DS, Zajko AB, Baron RL, Santaguida LA (1994). Hepatic artery stenosis and thrombosis in transplant recipients: Doppler diagnosis with resistive index and systolic acceleration time. Radiology.

